# Relationship between Kinesiophobia and Mobility, Impact of the Disease, and Fear of Falling in Women with and without Fibromyalgia: A Cross-Sectional Study

**DOI:** 10.3390/ijerph19148257

**Published:** 2022-07-06

**Authors:** Juan Luis Leon-Llamas, Alvaro Murillo-Garcia, Santos Villafaina, Francisco Javier Domínguez-Muñoz, Jesús Morenas, Narcis Gusi

**Affiliations:** 1 Universidad de Extremadura, Facultad de Ciencias del Deporte, Grupo de Investigación Actividad Física y Calidad de Vida (AFYCAV), 10003 Caceres, Spain; leonllamas@unex.es (J.L.L.-L.); svillafaina@unex.es (S.V.); fjdominguez@unex.es (F.J.D.-M.); ngusi@unex.es (N.G.); 2 Departamento de Desporto e Saúde, Escola de Saúde e Desenvolvimento Humano, Universidade de Évora, 7004-516 Évora, Portugal; 3 Universidad de Extremadura, Facultad de Ciencias del Deporte, Laboratorio de Aprendizaje y Control Motor, 10003 Caceres, Spain; jesusmorenas@unex.es; 4 International Institute for Innovation in Aging, University of Extremadura, 10003 Caceres, Spain

**Keywords:** activities of daily living, chronic pain, movement, pain, physical fitness

## Abstract

Background: Kinesiophobia is defined as fear of movement due to the painful experience of it. The main symptom of fibromyalgia is persistent and widespread pain associated with other symptoms. This study analyzes the kinesiophobia between women with fibromyalgia and apparently healthy women and investigates the relationship between kinesiophobia and physical fitness tests, fear of falling, and the impact of the fibromyalgia. Methods: Fifty-one women participated in this study were divided into two groups: (1) women with fibromyalgia and (2) apparently healthy women. Participants completed questionnaires to assess kinesiophobia, fear of falling, and the impact of fibromyalgia. Subsequently, participants completed the physical tests Timed Up and Go, 10-step stair ascent, and handgrip strength. Results: Women with fibromyalgia had significant differences in kinesiophobia and fear of falling compared to apparently healthy women. Similarly, performance in the physical tests was lower, except for the handgrip strength, which maintained similar values to the apparently healthy women. Significant relationships were found only in the fibromyalgia group between kinesiophobia, the impact of the disease, fear of falling, and the Timed Up and Go and 10-step stair ascent tests. Conclusions: Women with fibromyalgia showed higher kinesiophobia scores, worse performance in mobility tests, and higher fear of falling than apparently healthy women. Kinesiophobia score is related to Timed Up and Go performance, the 10-step stair ascent, the fear of falling, and the impact of the disease in women with fibromyalgia.

## 1. Introduction

Fibromyalgia (FM) is a complex disease characterized by chronic widespread pain, and it is often associated with fatigue, disrupted sleep, anxiety, depression, cognitive dysfunction, and poor physical fitness, among other symptoms [1,2]. These symptoms limit physical function, daily living activities, and emotional well-being [3,4], leading to a reduced health-related quality of life [5]. FM affects 0.2% to 6.6% of the general population, mainly in women over 50 years [6]. In Europe, the prevalence ranges from 2.9% to 4.7% of the general population and mainly occurs in women between 35 and 84 years [7]. In Spain, the global prevalence is estimated at 2.45%, of which women present 4.49% while men present 0.29% [8].

Due to reduced physical activity and a higher level of sedentarism, people with FM tend to present a relatively low physical condition [9]. Furthermore, people with FM commonly present balance problems that lead to a greater risk of suffering falls and a greater fear of falling [10]. All these physical function impairments could make the practice of physical activity difficult, increasing sedentary time and, consequently, the prevalence of overweight and obesity [11].

The Fear Avoidance Model proposes that pain-related fear activates mechanisms that lead to movement and activity avoidance, which, in the long-term, cause disability, disuse, and depression, entering a cycle that feedbacks and contributes to the maintenance and progression of the disability [12,13,14]. In FM, the fear of pain plays a crucial role in physical activity habits. In this regard, the initial level of pain associated with activities determines the level of physical activity a person with FM is willing to perform [15].

Kinesiophobia plays a fundamental role in the Fear Avoidance Model, since it consists of an irrational, excessive, and debilitating fear of movement arising from a sense of fragility or vulnerability due to injury or (re)injury [16]. Higher kinesiophobia has been consistently associated patients with chronic pain with higher levels of intensity of pain, disability [17], and lower quality of life [18]. Importantly, recent evidence highlighted the mediating role of kinesiophobia in the relationship between pain intensity and self-reported disability, suggesting the pivotal role of these factors in modulating the response to pain stimuli [19].

Previous studies have shown that kinesiophobia has a negative impact on activities of daily living in elder institutionalized people with chronic pain [20] as well as limited the physical activity status of patients with chronic low back pain [21]. Furthermore, kinesiophobia predicts mobility and balance in older people with low back pain [22]. Thus, the evaluation of kinesiophobia in clinical practice is relevant to recognize barriers that may affect compliance with rehabilitation programs [22]. In FM, 40% of patients exhibited high levels of fear of movement and avoidance behaviors toward physical activity [23]. In fact, treatment compliance in people with FM is affected by high pain levels, lower quality of life in the physical and social domains, and fear of movement [24].

In order to explore previous studies in the field of kinesiophobia and FM, a literature review was conducted in the PubMed database. The search string used was: “(kinesiophobia) AND (fibromyalgia)”. A total of 45 articles were retrieved on 19 June 2022 (date of the search). Of the 45 articles, most of them were focused on the effects of different therapies on different variables, including kinesiophobia. Only four studies explored the relationship between kinesiophobia and disability using questionnaires [25,26,27,28] that consider the physical activity performed (the last weeks or days) or how the person would feel performing an activity. However, these studies may lead to possible biases when participants remembered what they did or how they felt, since objective measures of physical fitness were not registered. In contrast, two studies explored the relationship between kinesiophobia and disability through the performance of physical fitness tests [3,29]. In this regard, Cigarán-Méndez et al. [29] did not show significant correlation between handgrip performance and kinesiophobia. In the same line, Huijnen, Verbunt, Meeus and Smeets [3] did not report significant correlation between climbing stair performance (three times up and down a ten-step-high stair at a self-chosen, comfortable speed) and kinesiophobia. None of these studies analyzed the relationship between kinesiophobia, number of falls, and fear of falling. Furthermore, an apparently healthy group that examined the relationship between kinesiophobia and disability was not included in the analyzed studies. In addition, of the previously mentioned six articles [3,25,26,27,28,29], only one study examined the relationship between kinesiophobia and the impact of the disease through the Revised Fibromyalgia Impact Questionnaire (FIQR) [25] (the updated version of the Fibromyalgia Impact Questionnaire (FIQ)). The rest either did so with FIQ or did not use it. 

In this sense, the present study aimed to contribute to the literature with new information by analyzing the relationship between kinesiophobia with the number of falls, fear of falling, the impact of FM, and physical fitness tests closely related to activities of daily living, such as the Timed Up and Go test (TUG) [30,31], the 10-step stair ascent test [32], or handgrip strength [33]. In addition, an apparently healthy group was incorporated. It is important from a clinical point of view since increasing the knowledge in this field can identify those activities that are considered challenging, allowing the design of more friendly activities to improve the management of this disease. Therefore, this study aimed: (1) to compare the kinesiophobia between women with FM and apparently healthy women and (2) to investigate the relationship between kinesiophobia and TUG, 10-step stair ascent test, handgrip strength, number of falls, fear of falling, and the impact of FM. We hypothesized that (1) kinesiophobia will be significantly higher in the FM group than in the apparently healthy group; (2) kinesiophobia will positively correlate with the performance in TUG and the 10-step stair ascent test but not correlate with the handgrip strength since the motor pattern required in the TUG and 10-step stair ascent tests are more challenging than handgrip strength; and (3) kinesiophobia will positively correlate with the fear of falling and the impact of the FM.

## 2. Materials and Methods

### 2.1. Participants

**Figure 1:**
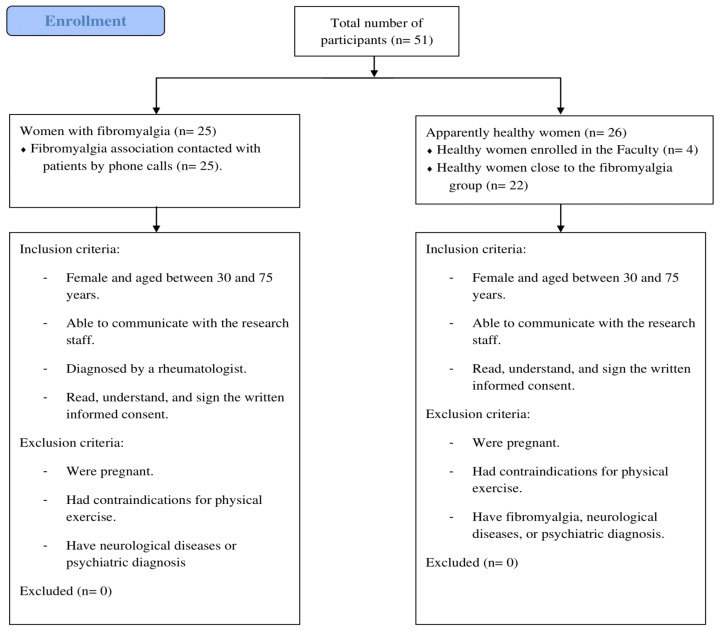
Flow chart of participants.

A total of fifty-one women (n = 25 women with FM; n = 26 apparently healthy women) from a convenience sample participated in this cross-sectional study. The sample size was calculated using G*Power 3.1 and based on the kinesiophobia results from Peinado-Rubia, et al. [26] (28.88 (7.11) for FM group) and Osumi et al. [34] (21.0 (4.1) for healthy participants). A total of 38 participants (19 both in the FM and the comparison group) were necessary to achieve a 99% statistical power. Assuming they did not participate in the measurements or some inconvenience occurred, 25% of the participants were added to each group. Therefore, 25 and 26 women were recruited for the FM group and the apparently healthy group, respectively. The flow chart of participants is depicted in Figure 1. Participants were recruited from a local association of FM in Cáceres (Spain), university facilities, and close contacts of FM patients by telephone calls. All the recruited participants fulfilled the following inclusion criteria: (a) female and aged between 30 and 75 years; (b) able to communicate with the research staff; (c) for the FM group, they have to be diagnosed by a rheumatologist according to the 2010 criteria established by the American College of Rheumatology [1]; and (d) have read, understood, and signed the written informed consent conforming to the updated Declaration of Helsinki. In addition, participants were excluded if they: (a) were pregnant, (b) had contraindications for physical exercise, and (c) have neurological diseases or psychiatric diagnosis. All procedures were approved by the University Research Ethics Committee (approval number: 62/2017), and all participants were verbally informed and gave written informed consent to participate in this study.

### 2.2. Procedure

First, age of the participants, height, weight, and body composition measured by bioimpedance (Tanita Body Composition Analyzer BC-418 MA, Tokyo, Japan) were assessed. Then, participants answered the Revised Fibromyalgia Impact Questionnaire (FIQR) [35], 11-item Tampa Scale of Kinesiophobia (TSK-11) [36], and Falls Efficacy Scale International (FES-I) [37].

After these first steps, participants carried out the physical Timed Up and Go (TUG) [38], 10-Step stair ascent [32], and handgrip strength [39] tests in the same day. Before starting the measurements, participants were informed about instructions and considerations for the correct development of the different physical fitness tests. The order of these tests were TUG, handgrip strength, and 10-Step stair ascent to prevent local fatigue in the upper and lower body. The procedure took place in the University facilities (Faculty of Sport Science, University of Extremadura, Cáceres) between January and March 2018.

### 2.3. Outcomes

(a) Impact of the disease, kinesiophobia, and fear of falling.

Revised Fibromyalgia Impact Questionnaire (FIQR). This extensively validated FM-specific tool is divided into three domains focusing on function, overall impact, and symptoms that evaluates the general impact of the disease from 0 to 100, where 0 indicates no impact and 100 indicates the highest impact [35]. The validated FIQR Spanish version was used in this study [40]. 

Tampa Scale of Kinesiophobia (TSK-11). TSK-11 is a robust predictor of disability based on fear of movement and injury [16,41]. This instrument has been used in patients with FM [28], chronic pain [42], and chronic low back pain [43], and it is used as a clinical diagnostic tool [28,36,44]. Kinesiophobia was measured by TSK 11-item [36] in its Spanish version [45]. Each of the 11 items has 4 answer options (1 = “strongly disagree”, 4 = “strongly agree”). The total score is calculated by adding the items. This total score can range from 11 to 44 points, with 11 indicating the lowest level of kinesiophobia and 44 the highest level of kinesiophobia. 

Falls Efficacy Scale International (FES-I). This tool is a self-report questionnaire developed and validated by the Prevention of Falls Network Europe (ProFaNE), providing information on the level of concern about falls for a range of activities of daily living inside and outside the home, providing excellent reliability and validity [37,46]. This questionnaire has been previously used in the FM population [47]. The FES-I contains 16 items scored on a four-point scale (1 = not at all concerned, 4 = very concerned), and a higher score is associated with a greater fear of falling [46]. The Spanish version was used in this study [48].

(b) Physical Fitness Tests

Timed Up and Go (TUG). This test has been previously used in the FM population, showing excellent reliability [38]. Participants have to get up from a chair without armrests, walk 3 m, turn around a cone, walk back, and sit down without using armrests. The time was measured using a manual stopwatch by one of the researchers.

The 10-Step stair ascent. In the FM population, this test has been previously used showing excellent reliability [32]. Participants have to climb 10 stairs with the instruction “as quickly and safely as you can”, but they cannot use handrails. The test starts with the subject in a static position. The recording time starts when the participant starts the movement and stops with the first step at the last stair. The time was measured using a manual stopwatch by one of the researchers.

Upper body muscular strength. A hand dynamometer (Takei TKK 5401 Digital Handgrip Dynamometer, Tokyo, Japan) was used to obtain the handgrip strength. This test has been previously used in FM patients [39,49]. The participant squeezes gradually and continuously for at least 2 s. The test was performed with the right and left hand twice, using a comfortable grip-span with the elbow fully extended and the palm of the hand perpendicular to the line of the shoulders in a standing position. The maximum score in kilograms for each hand was recorded.

The researcher who evaluated the physical fitness tests was blinded to group allocation. The participants were called to perform the physical tests on their assigned day. However, the researcher did not know the group to which the participants belonged, since he was only focused on evaluating.

### 2.4. Statistical Analysis

The SPSS statistical package (version 24.0; IBM Corp, Armonk, NY, USA) was used to analyze the data. Shapiro–Wilk test was conducted to analyze the distribution of the data. Variables with a *p*-value > 0.05 for each group (FM group and apparently healthy group) were required to consider a normal distribution. In this regard, non-parametric tests were conducted since some variables (age, number of falls, FES-I total score, 10-Step stair ascent, and handgrip strength) did not follow a Gaussian distribution. Furthermore, non-parametric tests are recommended in biomedical sciences [50].

Mann–Whitney U tests were performed to examine differences between groups in age, body mass index (BMI), time of the tests, strength, and total score tests. Furthermore, effect size [r] was calculated. It was classified as follows: 0.5 is a large effect, 0.3 is a medium effect, and 0.1 is a small effect [51].

Moreover, Spearman’s rho correlation analyses were used to evaluate the relationship between TSK-11, TUG, 10-Step stair ascent, handgrip strength, and FES-I.

The alpha level of significance was set at 0.05.

## 3. Results

The main characteristics of the participants and the differences between groups are shown in Table 1. The results indicated that the FM group had a moderate impact on the disease according to the classification by Bennett et al. [52].

The Mann–Whitney U test showed that the FM group reported higher kinesiophobia than the apparently healthy group, showing significant differences. The FM group also reported higher values of fear of falling than apparently healthy group measured through the FES-I. In the same line, FM group also reported a higher number of falls in the last year than apparently healthy group. Regarding physical fitness tests, lower levels of physical performance were found in the TUG and 10-Step stair ascent, showing significant differences when comparing the FM group with the apparently healthy group. These changes were not found in handgrip strength (see Table 1).

Correlation analyses between the values of kinesiophobia, the impact of FM, the fear of falling, and the performance developed in the physical tests were performed in the two groups. Only the values which correspond to the women with FM significantly correlated. However, significant correlation was not found for the handgrip strength for the apparently healthy group or the FM group. These results are shown in Table 2.

To obtain a more comprehensive analysis of the impact of fibromyalgia, the relationships between the score obtained in the TSK-11 and the specific questions that make up the domains of the FIQR are also shown. In this regard, within the “function” domain, a relationship was found with combing hair (r = 0.49, *p* = 0.013), lifting and carrying a bag full of groceries (r = 0.48, *p* = 0.015), and climbing stairs (r = 0.43, *p* = 0.032). A relationship was found with the overwhelming state of symptoms question inside the “overall impact” domain (r = 0.49, *p* = 0.012). Finally, in the “symptoms” domain, the pain question also showed a relationship with the score obtained in the TSK-11 (r = 0.49, *p* = 0.014). The rest of the questions did not show significant relationships.

## 4. Discussion

The present study aimed to evaluate the differences between apparently healthy women and women with FM in kinesiophobia. It also evaluated correlations between kinesiophobia and physical fitness tests (TUG, 10-step stair ascent test, and handgrip strength), fear of falling, and the impact of the FM. As we hypothesized, results showed that women with FM showed higher values of kinesiophobia and fear of falling than apparently healthy group. Furthermore, significant correlations were found in the FM group between kinesiophobia and impact of the disease, fear of falling, and performance in both TUG and 10-step stair ascent tests. Interestingly, a significant correlation was not found between kinesiophobia and handgrip strength in the FM group. Moreover, in the apparently healthy group, significant correlations were not found for any variable.

As commented above, our results showed that women with FM exhibited a higher level of kinesiophobia and fear of falling than the apparently healthy group. In this regard, our results are in line with a previous study that showed higher fear of falling levels in people with FM than in healthy controls [10]. These changes are probably due to the number of falls suffered [10], the symptomatology of FM, and other associated problems, including poor balance performance [10,53], postural instability [54], and poor functional performance [53]. Similarly, days with episodes of instability, kinesiophobia, and dizziness also explained more than half of the variance in the confidence in balance in FM patients [26]. Regarding kinesiophobia, previous studies found similar results [26,29]. However, Koçyiğit et al. [28] and Meeus et al. [55] found similar results but these investigations did not use the 11-item version of TSK. 

In this sense, kinesiophobia is relevant as it dramatically affects the levels of physical activity [42], the activities of daily living [20], and the quality of life [18]. This would make people with FM enter a vicious circle where fear induces fear of pain, and fear of pain leads to disability partly due to increased sedentary time [13,14,56]. Thus, physical activity interventions are quite relevant in this population since it is a way to break this vicious circle, reducing pain levels and increasing the health-related quality of life [57,58]. The assessment of kinesiophobia seems essential in clinical settings since it could act as a barrier to rehabilitation programs [22]. In this line, people with FM characterized by severe pain, high fear of movement, and a reduced quality of life in the physical and social domains reported reduced levels of adherence to treatment [24]. Nevertheless, future studies should establish cut-offs levels for TSK-11 in people with FM to make better interpretations.

Previous studies have shown that people with FM showed a physical impairment that could reduce their ability to perform activities of daily living [3,4]. In this regard, our results showed lower performance in the FM group than in the apparently healthy group in the TUG and the 10-step stair ascent tests. This information is in line with previous studies [32,53]. Moreover, these tests are relatively close to tasks which have to be performed in daily living where people with FM find difficulties [32,59]. Patients with FM generally show low handgrip strength levels compared to healthy subjects [39,49,60,61]. However, our results have shown similar handgrip strengths between the groups and are in line with a previous research [62]. It is possible that these differences may not have been found, since the apparently healthy group showed lower levels of strength in comparison with other studies that had a similar age range and used the same measurement instrument [49,60,61]. In this regard, it seems essential to homogenize the handgrip strength protocols used in order to be able to perform accurate comparisons between studies [63].

Considering the relationships between TSK-11 and the variables analyzed in this study, there are significant relationships in the FM group between kinesiophobia and fear of falling, TUG, 10-step stair ascent, and the impact of FM. These relationships can be explained through the Fear Avoidance Model [12,13]. Pain, the main symptom of FM, is associated with high values of kinesiophobia [12,18,42,56,64,65] and may induce changes in cortical networks that perceive and regulate motor functions [66]. The physical performance obtained in the TUG and 10-step stair ascent significantly correlated with kinesiophobia. This may be since kinesiophobia can cause the avoidance of physical activity during activities of daily living, inducing a vicious cycle that contributes to the maintenance and progression of the disease [67]. This relationship has also been previously observed in the TUG [29] but not in the 10-step stair ascent test [3], probably due to the comfortable pace used by the participants to climb the stairs. In addition, this vicious cycle can explain the relationship between kinesiophobia and the impact of the disease. In this regard, Russek et al. [25] also found significant correlation between kinesiophobia and the impact of the disease. Our study analyzed the specific questions of the domains that constitute the FIQR. Pain is related to the score obtained in the TSK-11, as are activities such as combing hair, lifting and carrying a bag full of groceries, climbing stairs, and the overwhelming state of symptoms. However, a significant correlation was not found between kinesiophobia and the handgrip strength test. This result is consistent with a previous study that did not find significant correlation between kinesiophobia and handgrip strength in people with FM [29]. Furthermore, Ishak et al. [22], in older people with low back pain, reported significant correlation between kinesiophobia and mobility and balance measured through the TUG but not with the handgrip strength. We hypothesized that this could be due to the context and the motor pattern involved in this task since it is a static test that does not involve a translation of the body in space, and therefore, women may not feel “fear” during this test. Finally, fear of falling is another variable related to kinesiophobia in the present study. This association may be due to the painful memory of having suffered a fall or the possible pain generated by a future fall based on the fear-avoidance model of falling and functional disability [68]. However, more research is needed to clarify these interesting aspects.

The current study has some limitations. First, the sample was relatively small and only composed of women, so we cannot generalize the results to men. The second limitation was this cross-sectional design study could not establish causality, requiring longitudinal research or a randomized controlled trial to clarify these questions. 

Furthermore, some recommendations for future research directions can emerge from this study. Firstly, it is necessary to continue using the 11-item TSK to assess kinesiophobia, establish cut-off points to identify the severity of kinesiophobia, and facilitate comparison between studies. Secondly, further research is needed into different treatment methods to improve physical performance, fear of falling, the impact of the disease, and kinesiophobia in this population through interventions that include larger samples based on size estimation, well-defined protocols, and long-term data. Similarly, future research should also include other significant factors associated with functioning, pain catastrophizing, and pain acceptance in FM. In this regard, a multidisciplinary approach is required due to the wide range of variables that could affect FM.

## 5. Conclusions

Women with FM showed higher kinesiophobia score, worse performance in mobility tests, and higher fear of falling than apparently healthy women. Kinesiophobia score is related to TUG performance, the 10-step stair ascent test, and the FES-I score and the impact of the disease in women with FM. However, this correlation was not observed in a physical fitness test with a less complex motor pattern such as handgrip strength test. The apparently healthy women did not show a significant correlation between kinesiophobia and any variables. The influence of kinesiophobia on conducting assessments in patients with chronic pain is relevant for healthcare professionals.

## Figures and Tables

**Table 1:** Differences between groups in demographic, total score questionnaires, and physical fitness tests.

Variable		Women with FM (n = 25) Mean (SD)	Apparently Healthy Women (n = 26) Mean (SD)	Value of Contrast	*p*-Value	Effect Size
Age (years)		56.36 (8.39)	54.69 (6.78)	−0.293	0.770	−0.041
Height (cm)		160.04 (7.02)	160.12 (6.92)	−0.661	0.509	−0.093
Weight (Kg)		68.64 (12.22)	62.99 (8.18)	−1.630	0.103	−0.228
BMI (Kg/m^2^)		26.79 (4.48)	24.72 (3.95)	−1.592	0.111	−0.222
Number of falls in the last year		1.52 (2.24)	0.42 (0.76)	−2.131	0.033	−0.298
FIQR		51.45 (17.71)	N.A.			
TSK-11		25.68 (6.54)	20.54 (6.20)	−2.394	0.017	−0.335
FES-I		28.22 (7.55)	18.88 (4.75)	−4.779	<0.001	−0.669
TUG (s)		6.42 (0.84)	5.92 (0.74)	−2.196	0.028	−0.308
10-Step stair ascent (s)		4.72 (1.04)	3.83 (0.68)	−3.241	0.001	−0.454
Handgrip strength (Kg)	L	23.03 (4.48)	24.32 (3.86)	−0.914	0.361	−0.128
	R	24.52 (4.69)	25.99 (3.89)	−1.159	0.246	−0.162

Abbreviations: FM, Fibromyalgia; BMI, Body Mass Index; FIQR, Revised Fibromyalgia Impact Questionnaire; TSK-11, 11-item Tampa Scale of Kinesiophobia; FES-I, Falls Efficacy Scale International; TUG, Timed Up and Go; SD, Standard Deviation; s, seconds; Kg, Kilograms; L, Left; R, Right; N.A., Not Applicable.

**Table 2:** Relationship between kinesiophobia, impact of FM, fear of falling, and physical fitness tests.

Variable			FIQR	FES-I	TUG	10-Step Stair Ascent	Handgrip Strength	
	Group						L	R
TSK-11	Women with FM	Correlation coefficient	0.458	0.543	0.441	0.470	−0.265	−0.178
		*p*-value	0.028	0.005	0.027	0.018	0.201	0.395
	Apparently healthy women	Correlation coefficient	N.A.	0.122	0.310	0.283	0.183	0.045
		*p*-value	N.A.	0.572	0.140	0.180	0.393	0.836

Abbreviations: FM, Fibromyalgia; FIQR, Revised Fibromyalgia Impact Questionnaire; TSK-11, 11-item Tampa Scale of Kinesiophobia; FES-I, Falls Efficacy Scale International; TUG, Timed Up and Go; L, Left, R, Right; N.A., Not Applicable.

## Data Availability

The datasets analyzed during the current study are available from the corresponding author on reasonable request.
